# The extracellular matrix molecule Collagen XVIII/CLE-1 affects neuronal dendritic spines

**DOI:** 10.17912/micropub.biology.001331

**Published:** 2024-11-05

**Authors:** Michele Lemons L, Hailey McKillop, Noelia Genao, Michael Francis M

**Affiliations:** 1 Department of Biological and Physical Sciences, Assumption University, Worcester, Massachusetts, United States; 2 Department of Neurobiology, University of Massachusetts Chan Medical School, Worcester, Massachusetts, United States

## Abstract

The extracellular matrix (ECM) is a rich collection of macromolecules that influences numerous cellular functions; however, its roles at neuronal synapses are not fully understood. Using dendritic spines of *Caenorhabditis elegans* GABAergic neurons as a model, we found that the ECM component Collagen XVIII/ CLE-1 is localized in close proximity to dendritic spines and is important for their normal development and maintenance. Specific expression of *cle-1* in GABAergic neurons partially rescued the reduction in spine number in *cle-1 ( cg120 )* mutants *.* Together, our findings suggest that Collagen XVIII/ CLE-1 regulates dendritic spines, in part through local CLE-1 deposition from GABAergic neurons.

**Figure 1. Collagen XVIII/ cle-1 affects dendritic spines:**
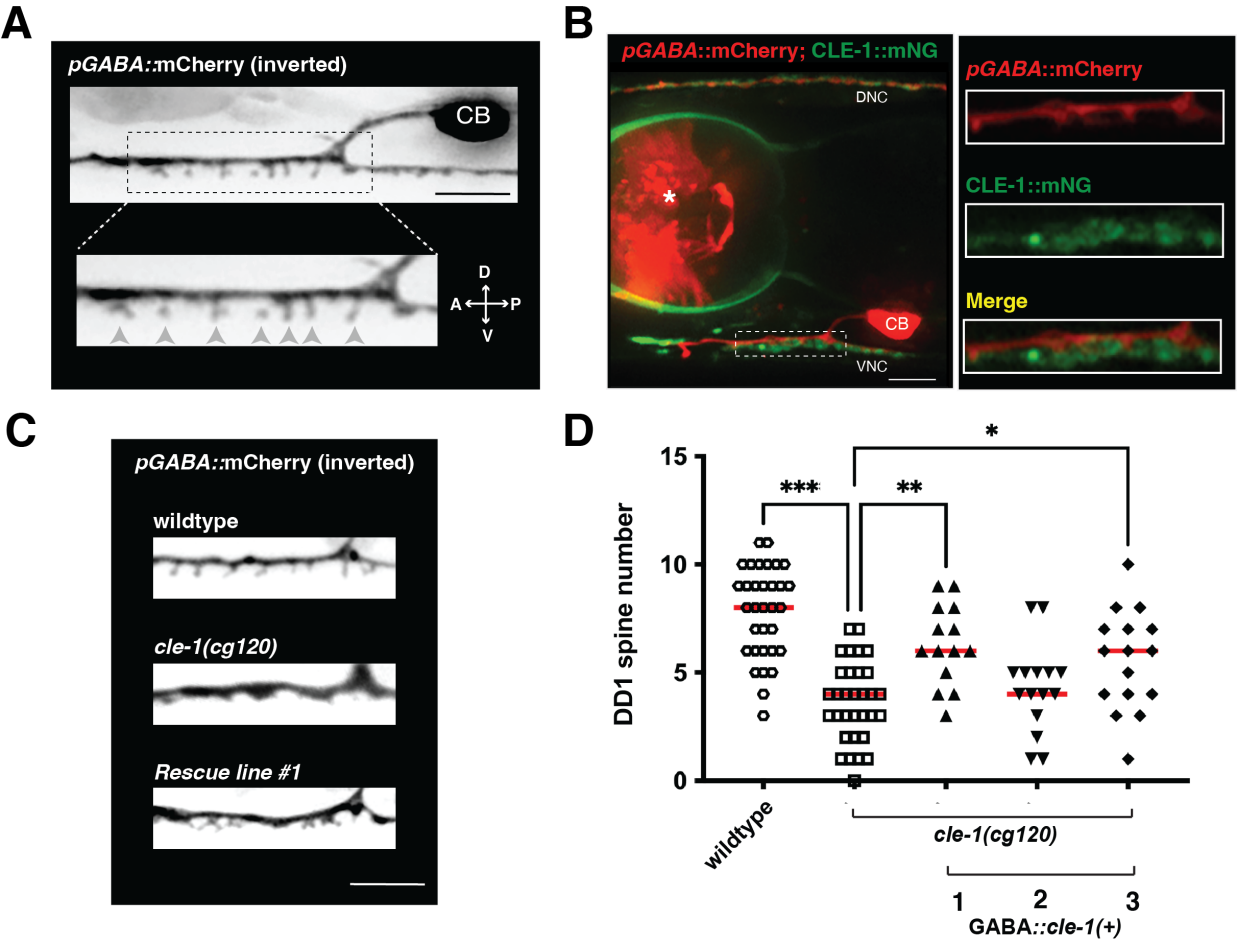
(A) Maximum projection confocal image of *C. elegans* GABAergic DD1 neuron (inverted LUT grey scale) labeled by mCherry. p *GABA::* mCherry refers to transgenic expression in GABA neurons using the *flp-13* promoter. Neuronal cell body (CB), dendritic shaft and dendritic spines of the DD1 neuron are shown. Dashed box highlights region enlarged in bottom panel, dendritic spines are marked by grey arrowheads. All images are from L4 animals and are oriented with anterior (A) to the left, and dorsal (D) towards the top. Scale bar, 5 μm. (B) Maximum projection confocal image showing endogenous mNG-tagged Collagen XVIII/ CLE-1 expression ( CLE-1 ::mNG, green) and DD GABAergic neurons labeled with *pGABA* ::mCherry (red). CLE-1 ::mNG labeling is in close proximity to DD1 dendritic spines (in dashed box), within the ventral nerve cord (VNC). Additional CLE-1 ::mNG expression is also observed near the dorsal nerve cord (DNC), and surrounding the pharyngeal bulb. Region within dashed box is enlarged in the right panel and shown as a single 0.3 μm confocal slice. Dendritic shaft and spines of DD1 labeled in red (top right panel, pGABA::mCherry), endogenous mNG-tagged CLE-1 expression labeled in green (middle panel, CLE-1 ::mNG), and merged images (bottom) are shown, revealing close proximity of CLE-1 ::mNG to DD1 dendritic spines. Scale bar, 5 μm. Asterisk indicates pharyngeal expression of co-injection marker. CB, cell body. (C) Representative inverted maximum projection confocal images showing DD1 dendritic spines from wildtype, *cle-1 ( cg120 )* , and rescue line #1. Rescue refers to specific expression wildtype *cle-1* cDNA in GABAergic neurons of *cle-1 ( cg120 )* mutant animals (GABA:: *cle-1 (+)* ). Scale bar, 5 μm. (D) Scatter plot showing quantification of spine number in DD1 neurons within a 15 µm region in wild type, *cle-1 ( cg120 )* mutants, and rescue strains. The number of DD1 dendritic spines was significantly decreased in *cle-1 ( cg120 )* mutants compared to wildtype. This mutant phenotype was partially and significantly rescued in two of three lines that expressed wildtype *cle-1* cDNA under a GABAergic promoter (GABA:: *cle-1 (+)* ) in a *cle-1 ( cg120 )* background. Red horizontal line indicates mean value. ANOVA with Tukey's multiple comparisons test. ***p<0.0001, ** p<0.001, *p<0.02.

## Description

The extracellular matrix (ECM) is a non-cellular network of macromolecules that serve as a scaffold to support surrounding cells and offer a rich network of biochemical and physiological signals. The ECM performs a wide range of functions including structural support, cell adhesion, cell migration, cell growth, cell polarity and cell survival (Frantz et al., 2010; Karamanos et al., 2021; Long and Huttner, 2019) . Several studies have investigated the role of ECM receptors at synapses but roles for specific ECM molecules in synapse development, positioning, and maintenance are not fully understood (Dityatev et al., 2010; Kerrisk et al., 2014; Levy et al., 2014; Senkov et al., 2014; Yang et al., 2023) .

To better understand how the ECM impacts neuronal synapses, we studied ECM interactions using recently characterized dendritic spines in dorsal-type D-class GABAergic neurons of the nematode *Caenorhabditis elegans* as a model (Alexander et al., 2023; Cuentas-Condori et al., 2019; He et al., 2015; Oliver et al., 2022; Philbrook et al., 2018) . Dendritic spines in *C. elegans* share many features with spines found on dendrites of mammalian neurons. These small actin-rich membrane protrusions house neurotransmitter receptors and other signaling machinery, and are the sites at which a majority of mammalian brain excitatory synapses are located (Hering and Sheng, 2001; Rochefort and Konnerth, 2012) . Dendritic spines play important roles in strengthening synaptic connections and signal transduction in mammals; however, the cellular machinery involved in formation and maintenance of these protrusions are not well understood. The transparent epithelium and well-characterized, largely invariant neuronal connectome of *C. elegans* enable *in vivo* studies of dendritic spines of the same single neuron across many animals, providing a unique opportunity to better understand roles of the ECM in dendrite biology.

We focused our studies on the ECM molecule Collagen XVIII/ CLE-1 that belongs to a specialized subset of ECM, the basement membrane, in part because prior single cell (sc)RNAseq studies indicated strong expression of *cle-1* in GABAergic motor neurons (Taylor et al., 2021) . Previous work suggests Collagen XVIII might be important at synapses between climbing fiber axons and Purkinje cell dendrites in the mouse cerebellum (Su et al., 2012) . In addition, mutations in Collagen XVIII/ *cle-1* disrupt GABAergic presynaptic organization (Ackley et al., 2003) and lead to ectopic presynaptic boutons in *C. elegans* (Qin et al., 2014) . Collectively, these studies support roles for Collagen XVIII/ CLE-1 in directing presynaptic organization, but contributions of CLE-1 to the organization of postsynaptic structures, such as dendritic spines, remain less well understood.

To investigate the potential role of Collagen XVIII/ CLE-1 at dendritic spines of GABAergic neurons, we used cell-specific expression of the red fluorescent protein mCherry to visualize GABAergic dendrites, as done previously (He et al., 2015; Oliver et al., 2022; Philbrook et al., 2018) . We imaged and analyzed dendritic spines of the DD1 GABAergic neuron ( Figure 1A ) due to its spatial isolation and largely invariant, easily identifiable location (immediately posterior and inferior to the second pharyngeal bulb). Similar to earlier work (He et al., 2015; Oliver et al., 2022; Philbrook et al., 2018) , we observed an average of 6.9 ± 0.3 (SEM) spines per 15 μm of DD1 GABAergic dendrite in wild-type larval stage 4 (L4) animals, the final larval stage before adulthood.

We examined the localization of Collagen XVIII/ CLE-1 relative to DD1 dendrites and associated spines using a genome edited strain where endogenous Collagen XVIII/ CLE-1 is tagged with mNeonGreen (mNG) (Keeley et al., 2020) . Consistent with previous reports (Keeley et al., 2020) , we noted strong CLE-1 ::mNG expression around the terminal bulb of the pharynx. We also observed significant CLE-1 ::mNG fluorescence near the dorsal and ventral nerve cords ( Figure 1B ), similar to previous findings using anti- CLE-1 antibodies (Ackley et al., 2001; Ackley et al., 2003) . Intriguingly, we also noted CLE-1 ::mNG fluorescent signal in close proximity to dendritic spines of the DD1 GABAergic motor neuron. In particular, we found that fluorescent signals associated with CLE-1 and spines were colocalized within a single 0.3 μm optical slice ( Figure 1B ), providing support for close proximity of CLE-1 to dendritic spines.

To investigate a potential role for CLE-1 in spine outgrowth or maintenance, we quantified the number of dendritic spines in L4 stage wild type and *cle-1 ( cg120 )* mutant animals. *cle-1 ( cg120 )* animals carry a deletion mutation that removes nearly the entire C-terminal noncollagenous NC1 domain (Ackley et al., 2001) . Truncated Collagen XVIII/ CLE-1 protein is detectable in *cg120* animals by immunohistochemistry albeit at lower levels than wildtype (Ackley et al., 2001) . Cell motility, axon patterning, and neuromuscular synapse organization are variably affected by this mutation (Ackley et al., 2001; Ackley et al., 2003; Kuo et al., 2001; Qin et al., 2014) . We found the number of dendritic spines on GABAergic DD1 neurons was strikingly reduced (by nearly 50%) in *cle-1 ( cg120 )* mutant animals compared to wildtype ( Figure 1C,D ). Specifically, the average number of spines in a 15 μm region of interest decreased from 6.9 ± 0.3 (SEM) in wild type to 3.4 ± 0.2 (SEM) in *cle-1 ( cg120 )* animals, suggesting Collagen XVIII/ CLE-1 plays an important role in dendritic spine formation or maintenance. Knowing the *cg120* allele may cause abnormalities in cell migration or axon extension (Ackley et al., 2001) , we restricted our spine quantification to DD1 neurons with normally positioned cell bodies, located ventral and slightly posterior to the terminal bulb of the pharynx, and with properly extended main dendrites that appeared qualitatively similar to that of wildtype. Thus, effects on cell body positioning or dendrite outgrowth are unlikely to account for the reduction in dendritic spines we observed.

Given the previous evidence for strong expression of *cle-1* in GABAergic neurons (Taylor et al., 2021) , we asked whether specific expression of wild-type *cle-1* in GABAergic neurons of *cle-1* mutants was sufficient to reverse the decrease in spine number we observed. We noted partial, but significant, rescue in two of three *cle-1* mutant lines that carried the *cle-1* rescuing transgene, demonstrating cell autonomous *cle-1* expression in GABAergic neurons is sufficient to partially normalize the outgrowth/maintenance of dendritic spines ( Figure 1C, D ). This finding is somewhat surprising since CLE-1 is expressed by a variety of cell types that likely contribute towards its distribution in the wildtype (Ackley et al., 2003; Graham et al., 1997; Taylor et al., 2021) . Our studies demonstrate that local deposition of CLE-1 from GABAergic neurons is at least partially sufficient to rescue postsynaptic spine defects.

In summary, our data show that the ECM molecule Collagen XVIII/ CLE-1 is expressed in close proximity to dendritic spines of GABAergic motor neurons. Our data also reveal that mutation of *cle-1* leads to a striking reduction in the number of dendritic spines on DD1 GABAergic neurons. Expression of wild-type *cle-1* solely in GABAergic motor neurons partially rescues this mutant phenotype, suggesting that deposition of CLE-1 by GABAergic neurons may be important for spine development or maturation. Prior work showed that the synaptic adhesion protein Neurexin/ NRX-1 is required for the maintenance of nascent spines during development (Oliver et al., 2022; Philbrook et al., 2018) . Our findings therefore also raise the interesting possibility that CLE-1 and NRX-1 may act in the same pathway to regulate spine organization.

## Methods

**Strains**

All strains are N2 Bristol strain derivatives (wildtype). Animals were maintained at room temperature (20-24 ^0^ C) on nematode growth media plates (NGM) seeded with *E. coli* strain OP50 . Some strains were provided by the *Caenorhabditis* Genetics Center (CGC), which is funded by the NIH Office of Research Infrastructure Programs (P40 ODO1O44). Transgenic strains were obtained by microinjection to achieve transformation (Mello et al., 1991) and identified by using co-injection markers. Integrated lines were produced with X-ray irradiation and outcrossed to wild type/ N2 Bristol eight times. Only L4 hermaphrodites were used in this study.

**Molecular Biology**

Plasmids were constructed using the two-slot Gateway Cloning system (Invitrogen) and confirmed by restriction digest and sequencing.

***cle-1* rescue constructs**

*cle-1* cDNA was synthesized from total RNA and amplified by RT-PCR using Superscript III Platinum Taq, assembled into pDest-16 (after digesting with NgoMIV + KpnI HF) using NEB HiFi Assembly to create pDest-377. pDest-377 was recombined by Gateway LR recombination (Invitrogen) with pENTR-3'- *unc-47* ( *unc-47* promoter) to generate pJR9 ( *P unc-47* :: *cle-1* cDNA) .

**Confocal microscopy and analysis**

Animals were immobilized in 0.3 M sodium azide on a 2% agarose pad. Images were obtained using a Yokogawa CSU-10 spinning disk confocal system on an upright Olympus BX51WI microscope with a Hammamatsu C9100-13 EMCCD camera and 63X oil immersion objective. Cobolt Calypso 491 nm and Jive 561 nm lasers were used for imaging. Image acquisition was using the software Volocity 4.3 (Perkin-Elmer). All images were obtained by imaging DD1 neurons in L4 hermaphrodites near the pharynx using identical imaging and laser settings for each marker. Z stack volumes were acquired using an objective-coupled Piezo (PI) at 0.3 µm/ z step. Image analysis was conducted using Fiji software (Version 2.14.0/1.54f) as described previously (He et al., 2015; Oliver et al., 2022; Philbrook et al., 2018) . Briefly, spines that were at least 0.2 μm in length were counted within a 15 μm ROI anterior to the DD1 cell soma. Spine length was determined by measuring from the base to the tip of each protrusion.

## Reagents

***C. elegans* strains used in this study:**

**Table:** 

**Strain name**	**Genotype**	**Description**	**Plasmid**	**Source**
IZ2680	*ufIs170*	DD-specific transcriptional reporter (red)	P *flp-13* ::mCherry (pAP31 @ 50ng/ul), co injection marker P *unc-122 :* :GFP (pPD97.98 @ 50ng/ul)	Francis lab
NK2322	*cle-1 ( qy22 ) *	CRISPR/Cas-9 mNG knock-in into C terminus of *cle-1* (green) (Keeley et al, 2020)		CGC
IZ4342	*cle-1 ( qy22 ); ufIs170*	CRISPR/Cas-9 mNG knock-in into C terminus of *cle-1* (green) (Keeley et al., 2020) , DD-specific transcriptional reporter (red)	P *flp-13* ::mCherry (pAP31 @ 50ng/ul), co injection marker P *unc-122* ::GFP (pPD97.98 @ 50ng/ul)	Francis lab
CH120	*cle-1 ( cg120 ) *	*cle-1* mutant with deletion in NC1 domain		CGC
IZ4345	*cle-1 ( cg120 ); ufIs170*	*cle-1* mutant with deletion in NC1 domain, DD-specific transcriptional reporter (red)	P *flp-13* ::mCherry (pAP31 @ 50ng/ul), co injection marker P *unc-122* ::GFP (pPD97.98 @ 50ng/ul)	Francis lab
IZ4491	*cle-1 ( cg120 ); ufIs170 ; ufEx1905*	*cle-1* mutant with deletion in NC1 domain, DD-specific transcriptional reporter (red), GABA-specific expression of wildtype *cle-1* cDNA, line 1	*ufEx1905* = P *unc-47* :: *cle-1* cDNA [pJR9 @25ng/µL], co injection marker P *lgc-11* ::mCherry [pBB107 @ 50 ng/µL]	Francis lab
IZ4493	*cle-1 ( cg120 ); ufIs170 ; ufEx1906*	*cle-1* mutant with deletion in NC1 domain, DD-specific transcriptional reporter (red), GABA-specific expression of wildtype *cle-1* cDNA, line 2	*ufEx1906* = P *unc-47 :: cle-1* cDNA [pJR9 @25ng/µL], co injection marker P *lgc-11* ::mCherry [pBB107 @ 50 ng/µL]	Francis lab
IZ4495	*cle-1 ( cg120 ); ufIs170 ; ufEx1908*	*cle-1* mutant with deletion in NC1 domain, DD-specific transcriptional reporter (red), GABA-specific expression of wildtype *cle-1* cDNA, line 3	*ufEx1908* = P *unc-47 :: cle-1* cDNA [pJR9 @25ng/µL], co injection marker P *lgc-11 ::* mCherry [pBB107 @ 50 ng/µL]	Francis lab

**Primers used to detect *cle-1 ( cg120 )***

Forward primer OMF3285 (GT Fwd primer spans intron 20 and exon 20 in *cle-1* Transcript): ACGATTCTAGATGgtcagttgg

Reverse primer OMF3286 (GT Rev primer in intron 20 on *cle-1* Transcript D): ccgtattccttctaccaccata

Generated 613 bp product from animals carrying allele for wildtype *cle-1*

Forward primer OMF3284 (GT Fwd primer in exon 17 based on *cle-1* Transcript D)

AGGAGACCTCCCAGAATACAAT

Reverse primer OMF3286 (GT Rev primer in intron 20 on *cle-1* Transcript D): ccgtattccttctaccaccata

Generated 432 bp product in animals carrying the *cg120* mutant allele.
